# Development and Initial Validation of the Italian Mood Scale (ITAMS) for Use in Sport and Exercise Contexts

**DOI:** 10.3389/fpsyg.2017.01483

**Published:** 2017-09-07

**Authors:** Alessandro Quartiroli, Peter C. Terry, Gerard J. Fogarty

**Affiliations:** ^1^Department of Psychology, University of Wisconsin – La Crosse, La Crosse Wisconsin, WI, United States; ^2^Division of Research and Innovation, University of Southern Queensland Toowoomba, QLD, Australia; ^3^Australian Centre for Sustainable Business and Development, University of Southern Queensland Toowoomba, QLD, Australia

**Keywords:** affect, emotions, measurement, multigroup analysis, BRUMS, sport, exercise, structural equation modeling

## Abstract

The current study presents initial validation statistics for the Italian Mood Scale (ITAMS), a culturally- and linguistically-validated Italian version of the Brunel Mood Scale (BRUMS: Terry and Lane, 2010). The ITAMS was administered to 950 sport participants (659 females), who ranged in age from 16 to 63 years (*M* = 25.03, *SD* = 7.62). In the first stage of the validation process, statistical procedures in Mplus were used to evaluate the measurement model. Multigroup exploratory structural equation modeling supported the hypothesized 6-factor measurement model for males and females separately and for the combined sample. Analysis of the scale scores using SPSS provided further support for the construct validity of the ITAMS with hypothesized relationships observed between ITAMS scores and measures of depression and affect. The development and validation of the ITAMS opens the way for mood-related research and sport or exercise interventions requiring mood assessments, in an Italian-language context.

## Introduction

Researchers have studied affective constructs such as emotion and mood in the context of sport and exercise domains for many decades (e.g., Morgan, 1980; Terry, 1995; Lazarus, 2000; Lane, 2007), producing a substantial body of knowledge related to the theory and measurement of the mood construct (e.g., McNair et al., 1971, 1992; Lane and Terry, 2000; Terry et al., 2003a). Mood is defined as “a set of feelings, ephemeral in nature, varying in intensity and duration, and usually involving more than one emotion” (Lane and Terry, 2000, p. 17). Several researchers have identified important conceptual distinctions between mood and emotion (e.g., Ekman, 1999; Beedie et al., 2005). Emotions are typically described as more intense, quicker to rise and shorter-lived than moods, and associated with more identifiable sources than mood. Conversely, moods are generally described as lower intensity, diffuse and enduring, without obvious antecedent cause, and less subject to conscious monitoring and control (Beedie et al., 2005). Further, moods tend to impart a more global influence over feelings, thoughts and behaviors, whereas emotions tend to engender the development of specific responses to situations requiring immediate action (Lane, 2007).

Although the mood construct is sometimes conceptualized as a unified psychological state, Lane (2007) stressed that moods involve a complex combination of feelings and that the interaction among mood dimensions, rather than any one aspect of mood in isolation, exerts influence on the behavior and ultimately on the performance of individuals. The relationship between affective states and performance has been studied extensively and is well-established in a variety of performance domains (e.g., Hanin, 1997; Beedie et al., 2000). Pleasant moods are generally associated with good performance whereas unpleasant moods tend to be associated with poor performance, and it is not unusual for athletes to attribute unsatisfactory performance to an inability to get into the “right mood” (Lane and Terry, 2016). However, research has demonstrated that unpleasant moods can sometimes facilitate good performance and pleasant moods may be associated with poor performance (Hanin, 1997; Beedie et al., 2000). Lane and Terry developed and tested a conceptual model to explain how different dimensions of mood interact to influence performance in sport and academic settings (Lane and Terry, 2000, 2005). In this model, depression is proposed to play a central role, although it is important to note that the term depression or depressed mood is used to describe unhappiness, dissatisfaction or dysphoria rather than a clinical condition.

Lane and Terry's model was based on the dimensions of mood as described by the Profile of Mood States (POMS; McNair et al., 1971). The POMS, initially developed to assess mood in clinical populations and subsequently in college populations, was later claimed to be also valid for use in sport and exercise settings (McNair et al., 1992) and has been used in several hundred sport-related studies (Lane, 2007). A number of abbreviated versions of the POMS have been developed, some of which have been criticized for using small validation samples or for incomplete validation processes (Lane, 2007). To address these limitations, Terry et al. developed and validated a 24-item short version of the POMS, designed primarily for use in sport and exercise domains, referred to as the Brunel Mood Scale (BRUMS; Terry et al., 1999, 2003a). The BRUMS includes six subscales corresponding to the underlying factors of Anger, Confusion, Depression, Fatigue, Tension, and Vigor. To account for the ephemeral nature of the mood construct, the BRUMS asks respondents to rate how they feel right now rather than retrospectively over the past week including today, as in the original POMS (Lane and Terry, 2000). The BRUMS has demonstrated satisfactory predictive, concurrent, criterion, and factorial validity as well as appropriate test-retest reliability (Terry et al., 1999, 2003a). Tables of normative data are available for athletes and non-athletes, covering both adolescents and adults (Terry et al., 2003a; Terry and Lane, 2010).

In addition to being used extensively with athlete samples, the BRUMS has been used in medical screening protocols (Galambos et al., 2005), to assess the risk of post-traumatic stress disorder among combat troops (van Wijk et al., 2013), to assess the risk of youth suicide (Gould et al., 2005), and to monitor cardiac rehabilitation patients (Sties et al., 2014). Although these uses include screening protocols in clinical environments (van Wijk et al., 2013), Lane and Terry (2016) stressed that the BRUMS should not be used as a stand-alone diagnostic tool.

When applying theories and models across cultures and nations, it is necessary to translate instruments, accounting for linguistic and cultural differences (Moustaka et al., 2010). The majority of published measures in psychology are produced in the English language, which provides a significant challenge for professionals working in other contexts and aiming to use appropriate, linguistically-validated scales (Rohlfs et al., 2008; Terry et al., 2012). Although researchers have long stressed the need to extend sport and exercise psychology research to cross-cultural settings (Duda and Allison, 1990), the need to develop cross-culturally validated measurement scales has become more acute due to the global expansion of the field (Quartiroli et al., 2014).

To address the need for greater cross-cultural generalizability of research findings, the BRUMS has been translated and validated in several languages, including Afrikaans (Terry et al., 2003b), Brazilian Portuguese (Rohlfs et al., 2008), French (Rouveix et al., 2006), Chinese (Zhang et al., 2014), Farsi (Terry et al., 2012), Hungarian and Italian (Lane et al., 2007), and Malay (Lan et al., 2012). Generally, a self-report measure is considered valid if it demonstrates content, factorial, criterion, and construct validity (American Psychological Association, 2014). Although an Italian translation exists of a 32-item, 8-factor version of the BRUMS, its authors noted that the scale suffered from “marginal fit indices, inconsistent factor loadings and relationships between mood states” (Lane et al., 2007, p. 121). We therefore judged that an Italian version of the more widely-used 24-item, 6-factor version of the BRUMS (Terry et al., 1999, 2003a) should be developed using a rigorous translation and validation process. This new measure is referred to as the Italian Mood Scale (ITAMS).

The primary aim of the study was to evaluate the factor structure of the ITAMS against the hypothesized 6-factor measurement model. The secondary aim of the study was to evaluate the concurrent validity of the ITAMS using the PANAS (Watson et al., 1988) and the Depression, Anxiety, and Stress Scale (DASS-21: Henry and Crawford, 2005) as external references. These scales were used because they featured in previous BRUMS validations (Terry et al., 2003a) and because valid and reliable Italian-language versions were available. Based on the previous findings of Terry et al. (1999, 2003a), we hypothesized positive relationships between the negatively-valenced ITAMS scales, the Depression scale of the DASS-21, and the PANAS Negative Affect (NA) scale. Moreover, we hypothesized that the ITAMS Vigor scale would correlate negatively with the NA scale and the DASS-21 Depression scale, and positively with the PANAS Positive Affect (PA) scale.

## Method

### Participants

Participants were 950 individuals residing in Italy who regularly engaged in recreational and/or competitive physical activities (284 males, 659 females, 7 not indicated; Age: range = 16–63 years, *M* = 25.03, *SD* = 7.62). A wide range of activities were represented, with the most common being football, track and field, swimming, wrestling, and volleyball.

### Measures

#### Italian mood scale (ITAMS)

**Figure 1:**
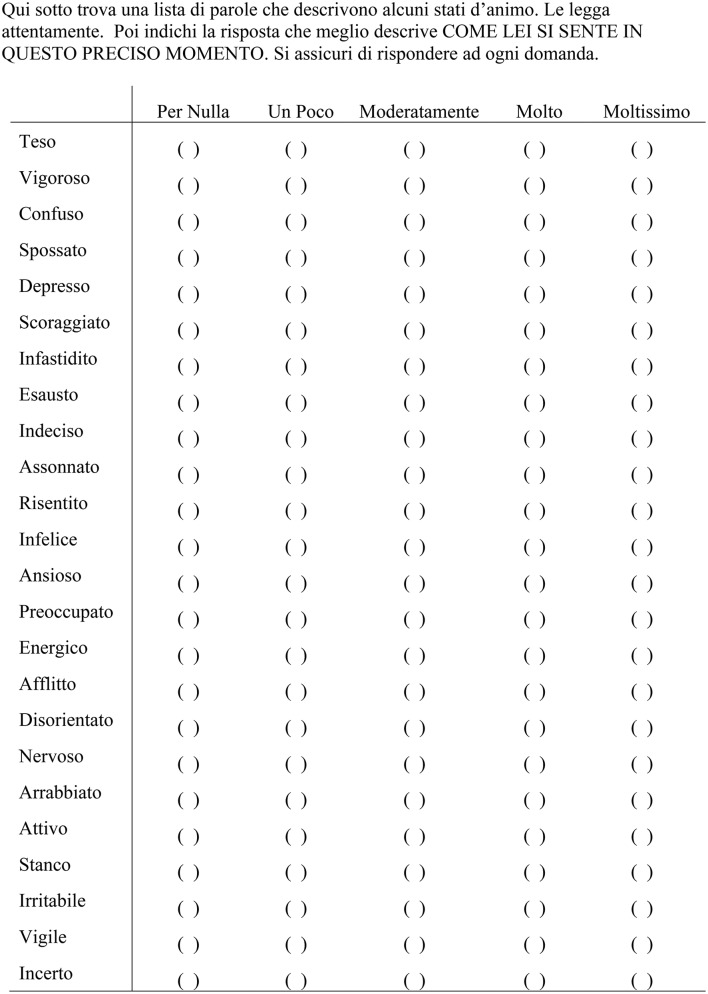
The Italian Mood Scale (ITAMS).

The ITAMS is an Italian-language version of the Brunel Mood Scale (BRUMS; Terry et al., 1999, 2003a; Terry and Lane, 2010). The ITAMS, which is reproduced in full in Figure 1, includes four mood descriptors to assess each of the six dimensions of mood; namely, Anger, Confusion, Depression, Fatigue, Tension, and Vigor. Participants indicated their feelings on a 5-point response scale ranging from 0 (*not at all/per nulla*) to 4 (*extremely/moltissimo*) rating “*how you feel right now/come lei si sente in questo preciso momento*” for each mood descriptor. Total scores were computed for each of the six subscales, where higher scores represented stronger endorsement of each mood dimension.

#### Translation of the brunel mood scale into Italian

The ITAMS was developed following a translation-back translation methodology (Brislin, 1970, 1986) by a group of bilingual (i.e., Italian, English) experts in sport, social, and clinical psychology. Three experts initially translated the BRUMS into Italian independently and then agreed upon a consensual version of the translation through discussion. Subsequently, three different experts in psychology initially independently and then consensually, translated the scale back into English. Finally, two psychology professionals, including an author of the original scale, checked the back translation to maximize the accuracy of the translation (Hambleton, 2005). Where discrepancies emerged between the original and the translated form of the BRUMS, the first two authors proposed possible alternatives. Minor modifications to wording and syntax were presented to the translation team to establish agreement and then implemented in the final version of the ITAMS.

#### Positive and negative affect schedule (PANAS)

Positive and negative affectivity were measured using a validated Italian-language version (Terracciano et al., 2003) of the PANAS (Watson et al., 1988). The PANAS has 10 positive and 10 negative affective descriptors rated on a 5-point Likert scale. The 1-week response timeframe was used. Watson and colleagues reported internal consistency reliabilities for PA and NA of 0.87 and 0.88, respectively. Eight-week test-retest reliabilities were 0.68 for PA and 0.71 for NA. Higher scores denoted higher levels of affectivity.

#### Depression anxiety stress scales-21 (DASS-21)

We used the validated Italian version (Bottesi et al., 2015) of the DASS-21 (Henry and Crawford, 2005), which has seven items rated on a 4-point Likert scale to assess each construct. Only the DASS-21 Depression scale was used in the present study. Participants indicated depressive symptoms over the previous week. Higher scores indicated higher levels of depression.

### Procedure

The study received approval from the Institutional Review Board for the Protection of Human Subjects at the first author's institution. An anonymous online survey hosted in a Qualtrics platform (Qualtrics, Provo, UT) was distributed via sport-specific social network groups and to college-aged students at four Italian higher education institutions using internal social networks. A four-phase follow-up procedure was implemented, sending reminder emails on the same networks at 3-week intervals (Dillman et al., 2014).

### Data analysis

The first step in the validation process involved fitting the 6-factor model to the covariance matrix representing the relationships among the 24 items. Exploratory structural equation modeling (ESEM) procedures were used to test model fit. These procedures have been advocated in cases where construct-relevant multidimensionality due to item fallibility or construct overlap makes it difficult to satisfy the independent cluster models restriction that underpins confirmatory factor analysis (Asparouhov and Muthén, 2009; Morin and Maïano, 2011; Myers, 2013). These procedures are available in Mplus 7.11 (Muthén and Muthén, 1998-2014) to generate fit indices to test the measurement model. They can also be used in multigroup analysis to assess factorial invariance across groups.

In addition to the χ^2^ test of exact fit, which is difficult to satisfy when the sample size is large, three fit indices were used to assess model fit. They were the comparative fit index (CFI) and the Tucker-Lewis Index (TLI), both of which require values above 0.90 and 0.95 for acceptable and excellent fit, respectively; and the root mean square error of approximation (RMSEA), which requires values less than 0.05 and 0.08, for close fit and reasonable fit, respectively (Marsh et al., 2004). The SPSS package was used for data screening, assessing subscale reliabilities, calculating inter-relationships among measured variables, and evaluating between-group differences.

## Results

### Data screening

Three cases were removed because they were missing more than 40% of their ITAMS data. The deletion of these three cases left a dataset of 947 respondents. One case was missing 29% of the ITAMS data but complete in other respects and was therefore retained. All other cases contained data for at least 22 out of the 24 ITAMS items. In other data screening procedures, inspection of item statistics showed that almost all items, Energetic (Item 10) and Active (Item 20) being the exceptions, were positively skewed. Mplus offers a solution to this problem that involves treating the items as ordered categorical (polytomous), rather than continuous variables. That option was pursued here.

### Model testing using exploratory structural equation modeling

Model solutions were estimated using diagonal weighted least squares with a mean-and-variance adjusted test statistic, operationalized as the WLSMV estimator in Mplus. The ESEM analyses were conducted using target rotation, which is appropriate when the expected factor structure is known, as in the present study (Browne, 2001). Given evidence that most of the mood scales are related (Terry et al., 2003a), oblique rotation was used. Although the chi-square test was significant, the approximate fit statistics indicated good fit: $\chi_{\left(147\right)}^{2}$ = 509.90, *p* < 0.001, CFI = 0.98, TLI = 0.97, RMSEA = 0.05 [90% CI = 0.05,0.06]. The standardized factor loadings are shown in Table 1.

**Table 1:** ESEM standardized factor loadings for the Italian Mood Scale (ITAMS).

**Items**	**Factors**					
	**Anger**	**Confusion**	**Depression**	**Fatigue**	**Tension**	**Vigor**
7. Annoyed (Infastidito)	0.56					
11. Bitter (Risentito)	0.44					
19. Angry (Arrabbiato)	0.78					
22. Bad tempered (Irritabile)	0.48					
3. Confused (Confuso)		0.63				
9. Mixed up (Indeciso)		0.86				
17. Muddled (Disorientato)		0.78				
24. Uncertain (Incerto)		0.90				
5. Depressed (Depresso)			0.75			
6. Downhearted (Scoraggiato)			0.60			
12. Unhappy (Infelice)			0.78			
16. Miserable (Afflitto)			0.57			
4. Worn out (Spossato)				0.67		
8. Exhausted (Esausto)				0.78		
10. Sleepy (Assonnato)				0.75		
21. Tired (Stanco)				0.94		
1. Panicky (Teso)					0.63	
13. Anxious (Ansioso)					1.08	
14. Worried (Preoccupato)					0.73	
18. Nervous (Nervoso)	0.46				0.61	
2. Lively (Vigoroso)						0.62
15. Energetic (Energico)						0.88
20. Active (Attivo)						0.88
23. Alert (Vigile)						0.44

*Loadings below 0.32 are not reported*.

We have used the convention of reporting loadings of.32 and above, where the factor accounts for at least 10% of the variance in the item (Tabachnick and Fidell, 2001). Loadings were all on target factors with no cross-loadings, with the sole exception of Item 18 (Nervous), which loaded on its target factor (Tension) but had a smaller cross-loading on Anger. This cross-loading can be explained by the colloquial use of *Nervoso* in Italian to indicate anger as well as nervousness. Item 13 (Anxious) had a loading above 1.00 on its target factor (Tension), which is a legitimate value when using oblique rotation because the loadings are regression coefficients, not correlations (Jöreskog, 1999). Scale inter-correlations were as expected and are presented in Table 2.

**Table 2:** Descriptive statistics, reliabilities, and correlations among measured variables.

**Variable**	***M***	***SD***	**α**	***Range***	**BRUMS *T-Score***	**1**	**2**	**3**	**4**	**5**	**6**	**7**	**8**	**9**	**10**
1. Age															
2. Gender						−0.20									
3. ITAMS-Anger	2.41	2.86	0.79	0–16	52–55	−0.06	0.01								
4. ITAMS-Confusion	2.72	3.15	0.85	0–16	50–53	−0.11	0.04	0.52							
5. ITAMS-Depression	2.24	2.88	0.85	0–16	53–57	−0.10	0.07	0.65	0.60						
6. ITAMS-Fatigue	5.03	3.57	0.82	0–16	53–56	−0.16	0.13	0.37	0.32	0.38					
7. ITAMS-Tension	3.92	3.61	0.86	0–16	45–48	−0.13	0.14	0.65	0.62	0.63	0.42				
8. ITAMS-Vigor	6.74	3.16	0.77	0–16	44–47	0.10	−0.20	−0.06	−0.12	−0.22	−0.31	−0.11			
9. PANAS-NA	15.14	6.01	0.90	10–50		−0.08	0.07	0.71	0.64	0.66	0.36	0.74	−0.08		
10. PANAS-PA	26.41	7.86	0.90	10-50		0.10	−0.16	−0.06	−0.09	−0.24	−0.21	−0.06	0.70	−0.02	
11. DASS-Depression	5.91	4.26	0.87	0–21		−0.12	0.14	0.42	0.49	0.62	0.28	0.47	−0.26	0.49	−0.26

### Multigroup analysis

Multigroup analysis was used to assess factorial invariance across males and females. The steps involved in multigroup analysis are slightly different with categorical indicators because thresholds are estimates rather than intercepts, and testing for factorial invariance involves two models rather than three. The first (default) model maintains equality of thresholds and factor loadings across groups, fixes the scale factor to one and the factor mean to zero in the first group and frees both parameters in the second group. In the second model, thresholds and factor loadings are free across groups, scale factors are one in both groups, and factor means are zero in both groups (Muthén, 2001). In the present study, the default model was sufficient to test equality of factor patterns, factor loadings, and means across males and females in this sample. The fit statistics for the baseline model were excellent for the male and female samples separately, as well as for the combined sample (see Table 3).

**Table 3:** Tests of measurement invariance across males and females.

**Model**						**90% CI for RMSEA**	
	**χ^2^**	**df**	**CFI**	**TLI**	**RMSEA**	**LL**	**UL**
1. Baseline Male	279.76	147	0.98	0.96	0.06	0.05	0.07
2. Baseline Female	356.01	147	0.99	0.97	0.05	0.05	0.05
3. Combined	687.69	468	0.99	0.99	0.03	0.03	0.04

*CFI, comparative fit index; TLI, Tucker-Lewis index; RMSEA, root mean square error of approximation; CI, confidence interval; LL, lower level; UL, upper level*.

### Descriptive statistics, reliabilities, correlations, and group differences

Following the successful fitting of the measurement models, the SPSS package was used to compute descriptive statistics and reliabilities for all scales (Table 2). Reliabilities for all scales were satisfactory, the lowest being 0.77 for the Vigor scale. There are no Italian norms for any of these measures, other than those generated through the present study. Hence, in the final column of Table 2, we have shown the T-score range from the BRUMS manual (Terry and Lane, 2010) that would capture the mean for our Italian sample. Nearly all participants in the current study (98%) were 18 years or older, so the T-scores were taken from the BRUMS table of normative data for adult athletes. It can be observed that the means for the Italian sample using the ITAMS were close to those reported for English-speaking adult athletes using the BRUMS.

The mean for the NA scale was close to the 16.00 reported by Crawford and Henry (2004) for a sample of English-speaking healthcare workers, while the PA mean of 26.41 was lower than the 31.31 reported for the PA scale. Finally, the mean of 5.91 for the DASS-21 Depression scale was higher than the 2.83 (Henry and Crawford, 2005) and the 2.35 (Sinclair et al., 2012) reported for English and US non-clinical samples, respectively.

Inter-correlations among the measured variables are shown in Table 2. Due to the large sample size, correlations above 0.09 were significant at the *p* < 0.01 level, so we considered these findings in terms of small (0.10 to 0.29), medium (0.30 to 0.49), and large (>0.50) effects (Cohen, 1992). Some of the correlations involving the two demographic variables, age and sex, represented small effects. Older athletes tended to be less confused, depressed, fatigued, and tense, and to have a more positive outlook and greater vigor. Female athletes tended to be more fatigued, tense, and depressed (on the DASS-21), and to report lower scores for vigor and positive affect. These outcomes partially replicated the findings of Terry et al. (2003b), who reported weak gender effects for tension, anger, and fatigue.

Correlations among the ITAMS subscales were as hypothesized. Using the Fisher r-to-z transformation to test for differences between the coefficients obtained here and those obtained by Terry et al. (2003a), the only differences were for the Tension subscale where the ITAMS coefficients were larger. Thus, for 10 out of a possible 15 comparisons, the correlations were the same (*p* < 0.01).

The three measures at the bottom of Table 2 provide an external frame of reference for the ITAMS scales. In four out of six cases, the correlations were in the predicted direction and represented large effects. The correlation between the Fatigue scale of the ITAMS and NA represented a medium effect and there was no association between the Vigor scale of the ITAMS and NA. The correlations between the ITAMS scales and PA were generally weaker, the exception being the Vigor scale of the ITAMS where the .70 correlation coefficient represented a large effect.

The last variable in Table 2 is the Depression scale from the DASS-21. It was expected to be positively associated with all the negatively-valenced ITAMS scales, most particularly with the Depression scale of the ITAMS. The results were as expected, with coefficients for four of the scales (Anger, Confusion, Depression, and Tension) representing medium effects, while the coefficient for Fatigue fell just short of the 0.30 criterion. Steiger's (1980) test of the difference between two dependent correlations, as implemented in Lee and Preacher's (2013) web utility, confirmed that the strongest correlation was between the Depression scales of the ITAMS and the DASS-21.

**Figure 2:**
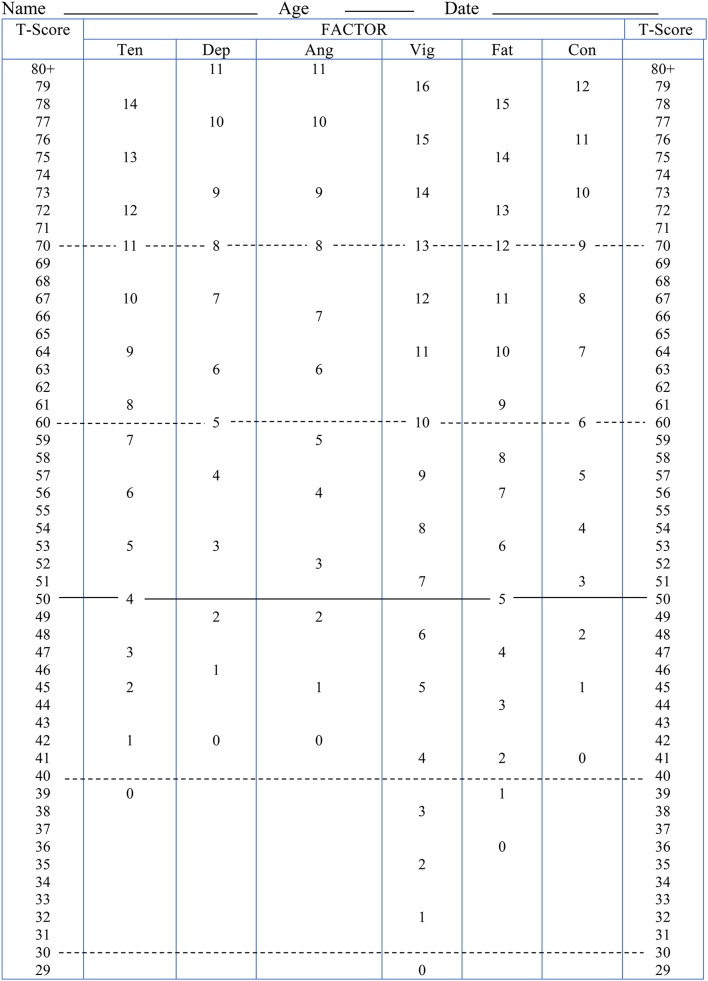
Italian Mood Scale (ITAMS) Profile Sheet.

A table of normative data for the ITAMS, based on the present sample, was generated using the recommendations of Thomas and Nelson (1996) and is presented in Table 4. To assist practitioners, normative data are also presented in the form of a mood profile sheet (Terry and Lane, 2010) in Figure 2.

**Table 4:** Standard scores for the Italian mood scale (*N* = 947).

**Raw Score**	**Anger**	**Confusion**	**Depression**	**Fatigue**	**Tension**	**Vigor**
0	42	41	42	36	39	29
1	45	45	46	39	42	32
2	49	48	49	41	45	35
3	52	51	53	44	47	38
4	56	54	57	47	50	41
5	59	57	60	50	53	45
6	63	60	63	53	56	48
7	66	64	67	56	59	51
8	70	67	70	58	61	54
9	73	70	73	61	64	57
10	77	73	77	64	67	60
11	80	76	80	67	70	64
12	84	79	84	70	72	67
13	87	82	87	72	75	70
14	91	86	91	75	78	73
15	94	89	95	78	81	76
16	98	92	98	81	84	79

## Discussion

We assessed the validity and internal consistency of the ITAMS by replicating the BRUMS validation process used by Terry et al. (1999, 2003a). Results indicated very good internal consistency of the six factors and excellent factorial validity. Factor inter-correlations were consistent with previous findings (Terry et al., 1999). As hypothesized, the ITAMS Depression scale was highly correlated with the other negative mood dimensions, while the ITAMS Vigor scale was inversely correlated with the five negative factors. ITAMS subscale means were comparable to those reported for adult athletes using the BRUMS (Terry and Lane, 2010). Concurrent validity of the ITAMS was supported via hypothesized relationships with the Italian versions of the PANAS (Terracciano et al., 2003) and the Depression scale of the DASS-21 (Bottesi et al., 2015). The five negatively-valenced ITAMS scales correlated with the NA and DASS-Depression scales, and were inversely correlated with the PA scale. The ITAMS Vigor scale was correlated with PA and inversely correlated with NA and DASS-Depression scales.

The measurement model of the ITAMS was confirmed via a multigroup analysis that confirmed factorial invariance across males and females. Fit statistics were very strong in the male, female, and combined samples. Although small gender differences in factor means were identified, they were not of sufficient magnitude to create model misfit, confirming that males and females conceptualize the mood construct similarly. Despite this conceptual consistency, there were differences in mood responses based on the gender and age of participants. Female athletes reported higher levels of fatigue, tension, depression (on the DASS-21), and lower levels of vigor and positive affect than their male counterparts. These results align with previous work by Terry et al. (2003b, 2012) and Rajkovic (2014) who found gender differences in mood responses among South African, Iranian, and Serbian samples. Moreover, older athletes reported lower confusion, depression, fatigue and tension scores, and higher vigor and PA scores than younger athletes, supporting previous findings (Zhang et al., 2014). Based on the comparability of responses to the BRUMS in paper-and-pencil and online versions (Terry and Lim, 2011), it is reasonable to assume that the ITAMS, although only administered online in our study, would show similar psychometric properties if administered in paper-and-pencil form. Future investigations should test this assumption.

Despite the demonstrated psychometric strength of the ITAMS, further exploration of its psychometric characteristics is necessary. First, although the ITAMS measurement model was supported, no analysis of its predictive validity was included in our study. Therefore, investigation of the relationship between mood and performance in Italian contexts is warranted to evaluate the predictive validity of the ITAMS. Second, test-retest reliability was not addressed in our study and hence further investigation is required to establish how well the ITAMS performs over multiple assessments. Given this lack of information about the psychometric consistency of the ITAMS over time, it is prudent to caution researchers and applied practitioners about drawing definitive conclusions from multiple assessments using the ITAMS until additional psychometric scrutiny has been completed. Third, due to the wide range of applications for mood scales, further investigation of the psychometric characteristics of the ITAMS among other populations of interest (e.g., youth, seniors, general population) and in other situational contexts (e.g., academic, business, medical, military) would be advantageous. Such research efforts can be informative in assessing the need for group-specific tables of normative data or whether the preliminary norms from the present study are equally relevant beyond the world of sport and exercise.

The ITAMS has a wide range of potential applications from both research and applied perspectives. From a research perspective, development of the ITAMS provides those researching in an Italian-language context with a mechanism for testing the central tenets of Lane and Terry's (2000) conceptual model of mood-performance relationships, and for replicating investigations of the predictive effective of mood assessments in sports such as karate (Terry and Slade, 1995) and swimming (Terry et al., 2004), The ITAMS also facilitates investigation of whether specific mood profiles, including the classic “iceberg” profile (Morgan, 1980) and more recently-identified profiles, such as the “inverse Everest,” “surface,” “submerged,” and “shark fin” profiles (Parsons-Smith et al., 2014), are evident among Italian speakers.

Other worthwhile lines of research include evaluating the efficacy of the ITAMS as part of medical screening protocols (Galambos et al., 2005), to assess risk of mental health issues (Gould et al., 2005; van Wijk et al., 2013), monitor cardiac rehabilitation patients (Sties et al., 2014), or as a quality of life index (Shin et al., 2016). Given the brevity of the ITAMS, it may serve a valuable role in mood research conducted in settings with limited time availability for data collection, such as prior to or between sport events. Further investigations of mood responses and mood regulation strategies among exercise participants, and comparisons between athletes and exercisers, would also appear to be salient.

From an applied perspective, the multiple uses for mood profiling in elite sport proposed by Terry (1995) are equally relevant in Italian-language contexts. Proposed uses for mood profiling include monitoring athlete responses to acclimatization and injury, as an indicator of general well-being, and as a catalyst for discussion. ITAMS assessments could also be used to gauge the effectiveness of mood regulation interventions (Lane et al., 2011) or as a self-monitoring tool to reduce risk of overtraining episodes (Lovell, 2004; Rohlfs et al., 2008). Given the global growth in the sport psychology profession (Quartiroli et al., 2014) and the associated need for practitioners to work with athletes from varied cultures (Vosloo and Quartiroli, 2014), cross-cultural validation of BRUMS translations such as the ITAMS help to support the delivery of sport psychology services across borders. The linguistic and cultural validation of the ITAMS is consistent with the quest for greater cultural sensitivity in applied sport psychology (Schinke and Hanrahan, 2009). Terry (2009), for example, described how belief systems engrained in cultural settings can play a significant role in how emotions are expressed and regulated. It is therefore important to further explore cultural influences that play a role in the experience, expression, regulation, and self-report of mood responses (Lan et al., 2012). Overall, our findings support the psychometric integrity of the ITAMS for use in Italian-language contexts.

## Author contributions

AQ, PT, and GF: contributed equally to the development and completion of the manuscript. AQ and PT: co-led the project and the manuscript development. GF: led the statistical analysis and contributed to the manuscript development.

### Conflict of interest statement

The authors declare that the research was conducted in the absence of any commercial or financial relationships that could be construed as a potential conflict of interest.
